# Behavioral Intervention versus Pharmacotherapy or Their Combinations in the Management of Overactive Bladder Dysfunction

**DOI:** 10.1155/2009/345324

**Published:** 2009-12-15

**Authors:** Khanh Tran, Robert M. Levin, Shaker A. Mousa

**Affiliations:** The Pharmaceutical Research Institute, Albany College of Pharmacy and Health Sciences, Rensselaer, NY 12144, USA

## Abstract

Overactive bladder syndrome (OAB) refers to individuals with the following symptoms: urinary urgency, increased urinary frequency, and urge incontinence. These symptoms are not life threatening but can cause embarrassment and significantly impact quality of life. There are numerous treatment options for OAB, including behavioral therapy, traditional pharmacological therapy or a combination of the two. These options are considered the mainstay of treatment for OAB. We carried out a comprehensive systematic review of the available literature on the effectiveness of behavioral intervention, anticholinergic drugs, and their combination in the management of adults with overactive bladder, with emphasis on results from clinical trials and primary literature. Each treatment intervention is efficacious, and the choice should be based on the patient's severity of symptoms, tolerability, compliance and satisfaction with the treatment. Based on available literature, management of OAB using a combination of behavioral therapy and drug intervention is the most efficacious in terms of patient satisfaction, perceived improvement, and reduction of bladder symptoms. It is also the most practical and cost effective for optimal management of patients with OAB. Pharmacological treatment, in addition to behavioral therapy, remains important in the management of adults with OAB syndrome.

## 1. Introduction

OAB is a common urinary dysfunction in both men and women, symptoms of which include urinary urgency, urinary frequency, nocturia, and urinary urge incontinence. OAB is estimated to occur in 33 million Americans or approximately 16.5% of the population [1–4]. In the long-term care setting, it is estimated that as many as half of all female residents suffer from incontinence [3, 4]. OAB is a serious medical and financial concern for several reasons. OAB is associated with medical and quality of life consequences that further compromise the health and well being of institutionalized and community-dwelling patients. Specifically, urinary incontinence (UI) has been associated with an increased risk of urinary tract infections, pressure ulcers, falls and fractures, which may severely compromise patient function and overall health [4–6]. Incontinence also consumes medical resources, staff, and provider time, which drives up the cost of care [6]. 

UI has been estimated to affect 20–33% of adults and greater percentages of the elderly [1–4]. UI is approximately twice as common among women than among men [5, 6]. Based on recent epidemiological studies, the overall prevalence of UI in women is 16.9%, which translates into an estimated 17.5 million women in the United States who suffer from this condition [7]. Prevalence increases with age, from 4.8% in women under the age of 25 to 30.9% in those over the age of 65 [7]. Elderly women that are institutionalized have a significantly higher prevalence of UI than those living at home, which is one of the contributing factors to institutionalization [1, 4]. Despite the high estimate of prevalence, however, it is believed that as many as 50% of all cases of UI may not be reported, and that individuals with UI do not always seek medical help [8–10].

UI has a substantial impact on physical and mental quality of life. The estimated total economic cost for UI was $19.5 billion in 2000 [5]. When indirect costs such as lost wages and productivity are included, the annual cost of incontinence increases to more than $26 billion, with institutional care accounting for more than 23% ($8.4 billion) [11]. In addition, the need for effective management strategies increases proportionally as the population ages and the proportion of elderly individuals aged 65 years and older increases.

The main symptom of OAB is urgency, or a sudden and compelling desire to pass urine which is difficult to defer [12]. Sometimes there is an involuntary leakage of urine with the feeling of urgency, which is called urge incontinence. The main complaints among individuals with OAB are the need to void too often during the day, and waking once or more per night to void [13]. In clinical practice, the need to void more than eight times during the day is considered daytime frequency, while waking from sleep more than once per night to void is considered nocturia.

Urgency and urge incontinence usually result from an involuntary increase in bladder pressure due to bladder smooth muscle over-activity during the filling phase of the micturition cycle. The pathophysiology of OAB remains unclear, but there are two widely accepted explanations. First, partial denervation of the detrusor muscle is believed to increase excitability secondary to changes in central nervous system (CNS) pathways that inhibit bladder activity [14]. Second, increased sensitivity of the sensory nerve endings in the bladder results in increased excitability of the detrusor muscle [14]. In either case, increased cholinergic stimulation of the detrusor occurs via postsynaptic muscarinic receptors [12].

The bladder contains both M_2_ and M_3_ subtypes of the muscarinic receptor. Even though the M_2_ subtype is more abundant, it is the M_3_ subtype that is mainly responsible for bladder contraction [15]. As a result, pharmacotherapy focuses mainly on the use of drugs with anticholinergic properties to block the parasympathetic acetycholine pathway and reduce the intensity of detrusor muscle contraction [16, 17]. By partially blocking the binding of acetycholine to detrusor muscarinic receptors, anticholinergic agents reduce the level of stimulation via parasympathetic neural impulses. Through this mechanism, anticholinergic therapy increases bladder capacity and delays the initial urge to void, thereby reducing the symptoms of OAB [12]. However, none of the current anticholinergic medications act specifically on muscarinic receptors in the bladder; they also act in other parts of the body, where they cause dry mouth, constipation, nausea, and other adverse effects.

Since OAB is a chronic condition, the goal in treatment is to reduce long-term health care costs, and increase quality of life (QOL) and patient satisfaction. In addition to antimuscarinic treatments, other current treatments for UI include behavioral modification, such as bladder training, fluid manipulation, scheduled toileting, pelvic muscle exercises, and surgical interventions. Often, pharmacological treatment and behavioral modification are combined [11, 18, 19].

Behavioral therapies are currently recommended as a first line therapy in the treatment of UI [20, 21]. Behavioral interventions are usually relatively inexpensive and easy to implement, but their effectiveness depends chiefly on patient motivation and compliance [22]. Thus, this type of treatment requires a high level of motivation and encouragement. The advantages of behavioral methods are improved central control of bladder function, avoidance of the mortality and morbidity of surgery, and no adverse drug reactions.

When nonpharmacological interventions have failed, drug therapy is commonly employed [10]. Drug treatment continues to play an important role in the management of women with UI, although many of the agents used have not been subjected to controlled clinical trials [23]. Based on a review of clinical studies reported in the literature, it is clear that there is no ideal drug, and there is a critical need for improved pharmacotherapy [24]. Current pharmacological approaches to improving the treatment of UI include delayed release formulations of existing oral agents, new pharmaceutical agents with greater specificity/selectivity, and alternative routes of administration. New generation pharmacological treatments, such as extended release (ER) tolterodine and transdermal oxybutynin, provide better or comparable efficacy with fewer adverse drug events [25–27].

## 2. Behavioral Therapies for Overactive Bladder and UI

As mentioned, behavioral therapies are considered the mainstay of treatment for urinary incontinence because they are noninvasive and can be initiated at the primary care level. However, the role that behavioral therapies play in the treatment of OAB has not been well addressed in the literature. Behavioral interventions include pelvic floor muscle training (PFMT), bladder training, and lifestyle modification. Lifestyle modifications for the management of OAB include smoking cessation, caffeine reduction, reduced alcohol consumption, weight loss, and limiting fluid intake. Several clinical trials have shown evidence of a relationship between obesity and OAB, suggesting that weight loss may significantly reduce episodes of urge incontinence [28–31].

There appears to be a dose-dependent relationship between caffeine and OAB. Patients with high caffeine intake (>400 mg/day) were 2.4 times more likely to experience detrusor overactivity [32]. Limiting fluid intake may also reduce frequency/urgency in OAB and improve patient quality of life [31, 33].

PFMT is most commonly used as a treatment for stress UI. The impact of PFMT appears to be multifactorial. In addition to the reflex inhibition of involuntary detrusor contraction, PFMT may provide the physical ability to voluntary contract the external sphincter and regain bladder control [34, 35]. Evidence to support the efficacy of PFMT for OAB is growing [36]. Patients with detrusor over activity who completed a PFMT program experienced a significant and clinically relevant reduction in daily UI episodes [35, 36]. There was also a significant decrease in urge score, defined as the frequency of leakage (0 = never to 4 = always) during 9 activities that can trigger urge incontinence.

Bladder training (BT) involves a systematic voiding regimen to lengthen the interval between voids until an acceptable pattern has been restored. Bladder diaries are used to establish maximum baseline voiding intervals, which are progressively increased by 15 to 30 minutes as tolerated. BT has been used for patients with stress incontinence, detrusor overactivity, or mixed incontinence, and clinical studies have demonstrated the effectiveness of BT in relieving the symptoms of UI [37, 38]. BT resulted in a significant reduction in UI episodes, including a significant decrease in volume of urine lost per UI episode. Despite this evidence of efficacy, however, the mechanism of BT's impact remains unclear, and there is little support for the common assertion that BT increases bladder capacity. McClish and coworkers reported a significant reduction in UI but no significant change in urodynamic parameters after 6 weeks of BT, while Else and colleagues found a significant increase in capacity at first sensation to void but not in maximum cystometric capacity [39, 40]. Regardless of mechanism, these studies demonstrate the effectiveness of BT in reducing UI.

Although behavioral modification has proven to be effective in reducing the symptoms of UI and OAB, it is not considered a cure. In addition, there have been no head-to-head trials of different behavioral interventions which would support one type over another. Increasingly, the emphasis in treatment of OAB is on combined behavioral and pharmacologic therapy.

## 3. Pharmacotherapy for OAB and UI

Anticholinergic drugs have been available for the treatment of OAB for years and their use is widespread in clinical practice. The number of anticholinergics on the market is increasing, and their effectiveness has been assessed in many studies [41–45]. There are questions regarding the role of anticholinergics in different patient groups such as the elderly, men, women, and patients with comorbidities, as well as the best route of administration. Despite these uncertainties, anticholinergics are increasingly being used in primary and secondary settings, particularly for the treatment of urge incontinence [46].

The International Consultation on Incontinence recommends anticholinergics as a first line pharmacotherapy in urge incontinence for men, women, the elderly, and patients with neurogenic detrusor overactivity. The main disadvantage to the use of this class of drug is the side effect profile. Newer generation anticholinergic medications have fewer side effects as compared to the older generations, but dry mouth is still a common adverse effect reported in almost one third of patients receiving these drugs [47]. Also, there are few studies that report the long-term effectiveness of these drugs [17].

### 3.1. Oxybutynin

Oxybutynin is an anticholinergic agent that has antimuscarinic, antispasmodic, and potential local anesthetic effects [48, 49]. It has been in widespread clinical use since 1975 and has been the most extensively studied in this class of drugs by clinicians. It is available in immediate-release (Ditropan), extended-release (Ditropan XL), and transdermal (Oxytrol) forms. Oxybutynin has been shown to have a high affinity for muscarinic receptors in the bladder, with a higher affinity for M_1_ and M_3_ receptors than M_2_ [50, 51]. The effectiveness of different forms of oxybutynin in the management of patients with detrusor overactivity is well documented.

A 2007 study by Stohrere and colleagues found that immediate release (IR) oxybutynin increased maximum cytometric capacity from 164 to 298 mL and decreased maximum detrusor pressure from 68.6 to 43.1 cm H_2_O in patients with detrusor overactivity [52]. A trial of extended-release oxybutynin (oxybutynin XL) showed that cystometric bladder capacity increased from 274 to 380 mL and incontinence episodes dropped from 13 to 6 per week, even though postvoid residual urine increased in patients treated with oxybutynin XL [53].

Oxybutynin XL has a less severe dry mouth side effect than oxybutynin IR and is more commonly used in practice [53]. In one study, the incidence of moderate to severe dry mouth in patients on oxybutynin XL was 23%, with only 1.6% of patients discontinuing the medication due to adverse effects [54]. A follow-up study that evaluated oxybutynin XL in patients with comorbidities (spinal cord injury, multiple sclerosis, and Parkinsonism) confirmed the improvement in urodynamic parameters [55]. The latter study concluded that the administration of up to 30 mg/day of oxybutynin XL was both efficacious and safe in these populations. Recently it was demonstrated that the use of oxybutynin XL in the elderly may impair recent memory [56].

In order to maximize efficacy and minimize adverse effects, an oxybutynin transdermal delivery system (oxybutynin-TDS) was recently developed and compared with oxybutynin XL. Oxybutynin—TDS increased cystomic capacity from 247 mL to 395 mL, increased reflex volume from 190 mL to 249 mL, and decreased maximum detrusor pressure from 61.2 to 37.8 cm H_2_O [57]. A maximum dose of 11.7 mg per day was found to be well tolerated by patients while improving continence. In a related study comparing oxybutynin-TDS to tolterodine, the most common side effect was application site reaction (14%), and the incidence of dry mouth was reduced to 4.1% as compared to the tolterodine treatment group (7.3%) [58].

### 3.2. Tolterodine

Tolterodine is a competitive muscarinic receptor antagonist with relative functional selectivity for bladder muscarinic receptors [59]. While the drug shows no specificity for receptor subtypes, it does appear to target the bladder over the salivary gland [60]. Tolterodine is available in immediate- and extended- release forms, and both formulations showed good efficacy for symptoms of OAB in a large study population [61]. However, extended-release is preferred by both clinicians and patients due to a less severe side effect profile [62]. In a randomized controlled study of tolterodine XL 4 mg/day in patients with urge incontinence and urinary frequency, no differences were found in terms of efficacy, tolerability, or safety between older and younger patients [61].

A recent trial comparing tolterodine XL to tolterodine IR and placebo found tolterodine XL to be significantly more effective [63]. In addition to increased efficacy, tolterodine XL has been shown to have better tolerability. In a double-blind, multicenter, randomized placebo controlled trial, tolterodine XL was found to be 18% more effective in reducing episodes of urge incontinence, with a 23% lower incidence of dry mouth [64]. The efficacy of tolterodine XL was confirmed in several clinical trials, including the OPERA (Overactive bladder: Performance of Extended Release Agents), OBJECT (Overactive Bladder: Judging Effective Control and Treatment), and ACET (Antimuscarinic Clinical Effectiveness Trial) studies [65–67]. The safety of tolterodine has also been confirmed, and at the recommended daily dose, the incidence of adverse events was no different than placebo [68].

In a comparison of the safety and efficacy of tolterodine versus oxybutynin, clinical efficacy was similar for both drugs; however oxybutynin had higher withdrawal rates and a higher incidence of dry mouth [45, 69].

### 3.3. Trospium Chloride

Trospium is a smooth muscle relaxant that is nonselective for muscarinic receptor subtypes [70]. It is a quaternary ammonium compound that was recently approved by the US Food and Drug Administration (FDA) for the treatment of OAB [71]. Trospium was initially provided as a single 20 mg dose for twice daily use; it has more recently been approved in a 60 mg once daily formulation. In multiple efficacy trials in OAB patients, trospium produced significant improvements in maximum detrusor pressure, maximum cystometric capacity, and bladder volume at first involuntary contraction [72–75]. In a study testing double daily dosages of trospium chloride (90 mg) in a mixed incontinence patient population that did not respond well with a standard dose, treatment with a double dose yielded improvements in reflex volume, cystometric capacity, and detrusor pressure as compared with a standard dose (45 mg) [73]. When an even higher dosage (90 mg/day) of trospium chloride was tested, it was reported that patients who were not responsive to the standard dose (45 mg) could be safely treated with up to three times the dose of trospium/day [76]. Similar to solifenacin (see below), trospium chloride may have a lower risk of CNS side effects because of its lower predilection to cross the blood-brain barrier [77]. It also has the added benefit of being metabolized by the kidneys rather than the cytochrome P450 system, thereby having fewer interactions with other medications [77]. Thus, this therapy may be beneficial for patients who are elderly and/or receiving multiple medications.

Trospium has been compared to oxybutynin in a randomized, multicenter trial [78]. The trial found that with both agents, there was a significant increase in bladder capacity, a decrease in maximum voiding detrusor pressure and a significant increase in compliance as compared to placebo, although there were no statistically significant differences between the two treatment groups [78]. However, patients taking trospium had a lower incidence of dry mouth and were less likely to withdraw as compared to the oxybutynin group [78].

### 3.4. Solifenacin

Solifenacin is an antimuscarinic agent and a potent M_3_ receptor antagonist that has selectivity for the M_3_ over the M_2_ receptor [79]. It also has much higher potency against the M_3_ receptor in smooth muscle than it does against the M_3_ receptor in the salivary gland [79]. Solifenacin efficacy has been proven in multiple trials in OAB patients [80–82]. For instance, phase III studies with solifenacin (5 and 10 mg) consistently showed it to be more effective than placebo for all OAB variables measured [80]. A recent SUNRISE study showed that solifenacin was significantly more effective than placebo in reducing the mean number of episodes of severe urgency with or without incontinence per 24 hours, improved urgency as early as day 3 of treatment, and was well tolerated [83]. Importantly, in contrast to oxybutynin, it is postulated that solifenacin is very unlikely to cross the blood brain barrier due to its biochemistry, and no cognitive impairment associated with this drug has been reported [84].

In a direct comparison between solifenacin in 5 and 10 mg doses and tolterodine XL in a randomized, double-blind trial, solifenacin was at least as effective as tolterodine XL in reducing micturition frequency, and it was significantly more effective than tolterodine XL in improving most other OAB variables [81]. In a meta-analysis of seven placebo-controlled OAB studies, the mean and median reductions in the number of urgency episodes with solifenacin were greater than with several other agents such as oxybutynin, tolterodine, trospium, and darifenacin [85]. These studies suggest that solifenacin is an effective treatment for OAB with the most commonly reported adverse events being dry mouth, constipation, and blurred vision.

### 3.5. Darifenacin

Darifenacin is a highly selective M_3_ receptor antagonist that was approved in 2004 for the treatment of OAB. It has a fivefold higher affinity for the M_3_ receptor relative to the M_1_ receptor [79]. This M_3_-selective receptor antagonist is potentially more bladder-specific, with a reduced tendency for anticholinergic side effects [86]. Darifenacin is available in 7.5 mg/day and 15 mg/day formulations. The drug is associated with relatively high rates of constipation (14.4% for darifenacin 7.5 mg daily) [87]. The efficacy of darifenacin has been investigated in several multicenter studies [88–91]. Darifenacin was significantly superior to placebo for improvements in micturition frequency, bladder capacity, frequency of urgency, severity of urgency, and number of incontinence episodes leading to a change in clothing or pads. In a comparison of darifenacin to oxybutynin, both drugs had similar efficacy; however, darifenacin had a less severe side effect profile than oxybutynin [92].

The most common adverse events associated with darifenacin are mild to moderate dry mouth and constipation, with a CNS and cardiac safety profile. Darifenacin has also been tested in patients who expressed dissatisfaction with prior OAB treatment with oxybutynin ER or tolterodine ER due to insufficient efficacy, tolerability problems, or both. The results of this trial showed significant improvements in OAB symptoms with darifenacin [86]. Long-term studies have shown that there are high levels of persistence with darifenacin therapy and well-maintained treatment benefits (over 2 years in duration) [93, 99]. Darifenacin minimizes the risk of functional side effects due to blockade of other muscarinic subtypes, such as M_1_-mediated cognitive impairment [94]. This aspect of darifenacin therapy becomes important in the treatment of elderly populations who may be more susceptible to cognitive impairment and CNS effects. This patient population is at an increased risk of advanced age conditions such as diabetes, Alzheimer's, or multiple sclerosis. One analysis by Foote in patients aged 65 years or more found that darifenacin had a low incidence of nervous system effects (somnolence, dizziness), but showed a significant improvement in all OAB scores.

## 4. Behavioral and Drug Therapy for OAB

A combination of anticholinergic agents and behavioral interventions is a safe and effective first-line treatment for OAB [8, 20, 21, 95]. Most patients do not achieve complete continence with either therapy alone. In addition, long-term medication adherence can be difficult due to side effect profiles. Adding behavioral training to pharmacologic treatment is an appealing approach to improving outcomes and to possibly permitting discontinuation of drug therapy. Moreover, anticholinergics may work synergistically with behavioral intervention, due to the different mechanisms involved [96]. Many trials have shown the synergistic effect of these two interventions (Table 1).

The Mattiasson trial showed that a combination of tolterodine and BT significantly reduced void frequency and increased volume per void as compared to tolterodine alone [97]. Furthermore, 76% of the patients on tolterodine and BT reported an improvement in their bladder symptoms relative to baseline as compared with 71% in the tolterodine group [97]. This finding is consistent with a previous trial showing that BT significantly increases the efficacy of tolterodine for reducing voiding frequency and increasing volume voided [98].

Clinical data has also shown that combination therapy is associated with significantly fewer incontinent episodes, better quality of life, and greater treatment satisfaction as compared to nonpharmacologic intervention alone or drug treatment alone [100]. However, the effects of each of the 3 interventions were similar 3 months after treatment, suggesting that the nature of the treatment may not be as important as having a structured intervention program that includes education, counseling, and frequent monitoring of the treatment [100].

## 5. Summary and Conclusions

The choice of therapy for treating patients with OAB will be based on many factors, including prognosis of underlying disease, mental status, age, education, motivation, and mobility, and should be tailored to the individual. A conservative management regime would begin with simple therapy (behavioral intervention) before proceeding to pharmacological treatment. Due to the complexities of the disease and individual variability, no single treatment for OAB exists. Rather, several treatments are typically begun and modified in order to meet the patient's desired therapeutic goal. The main goal in treating OAB is the preservation of upper urinary tract function and improvement of the patient's troubling urinary symptoms.

The prevalence of OAB is expected to increase in the future with the increasing number of elderly individuals in the population, emphasizing the need for methods to successfully manage OAB. Given the effectiveness of behavioral therapy, such as the absence of side effects, low cost, and ease of implementation in the outpatient setting, this approach is generally recommend as the first choice for OAB in the elderly.

The effectiveness of drug therapy and behavioral interventions for reducing urinary incontinence is well established [96–98, 100, 101]. There is strong evidence that a combination of approaches is useful in helping patients with OAB reduce incontinence episodes in certain settings. However, although combination therapy may be beneficial for reducing incontinence frequency during active treatment, a stepped approach, in which a single intervention is initiated first and then a second therapy is added for patients who do not achieve a satisfactory outcome, may be more practical and cost effective for optimal management of patients with OAB.

## Figures and Tables

**Table 1:** Impact of various interventions in OAB patients.

Intervention	Outcome	References
Weight loss	Obesity associated with OAB, moderate weight loss associated with improvement in urge UI	[28–31]
		
Caffeine restriction	Relationship between caffeine intake and OAB in men/women not significant: impact of caffeine on OAB may be dose dependent	[32]
		
Fluid restriction	Limiting fluid intake may reduce frequency/urgency in OAB, improve quality of life	[31, 33]
		
Bladder training	Used for patients with stress incontinence, detrusor overactivity, or mixed incontinence: lengthen the interval between voids, significant reduction in UI episodes in 3-day bladder diaries, and significant decrease in volume of urine lost	[37, 38]
		
Pelvic floor muscle training	Common treatment of stress UI: significant reduction of daily UI frequency, night time UI frequency, and frequency of leakage	[35, 36]
		
Oxybutynin (immediate release, extended release, transdermal)	Increased maximum cytometric capacity and decreased maximum detrusor pressure in patients with detrusor overactivity	[52–54, 57]
		
Tolterodine (immediate, extended-release forms)	Increased maximum detrusor pressure, reflex volume, and cystometric capacity but lower withdrawal rates and less incidence of dry mouth compared to oxybutynin	[65–69]
		
Trospium chloride	Produced significant improvement in maximum detrusor pressure, maximum cystometric capacity, and bladder volume; beneficial for patients who are elderly and/or receiving multiple medications due to its lower predilection to cross the blood-brain barrier	[72–75, 77]
		
Solifenacin	Effective in reducing micturition frequency, reducing the mean number of episodes of severe urgency with or without incontinence per 24 hours and improving urgency at day 3 of treatment; improves most other OAB variables	[80–84]
		
Darifenacin	Significantly superior to placebo for improving micturition frequency, bladder capacity, frequency of urgency, severity of urgency, and number of incontinence episodes leading to a change in clothing or pads; can be used for elderly population due to lower nervous system effects	[87–90, 93]
		
Behavior and drug therapies	Combination therapy produced added benefit in terms of patient satisfaction, perceived improvement and reduction of bladder symptoms; anticholinergic may work synergistically with behavioral intervention because of the different mechanisms involved	[8, 20, 21, 94–98]
